# Fisetin decreases TET1 activity and CCNY/CDK16 promoter 5hmC levels to inhibit the proliferation and invasion of renal cancer stem cell

**DOI:** 10.1111/jcmm.14010

**Published:** 2018-11-08

**Authors:** Yibing Si, Junfeng Liu, Hongliang Shen, Chen Zhang, Yuanhao Wu, Yongyi Huang, Zhangbin Gong, Jun Xue, Te Liu

**Affiliations:** ^1^ Division of Nephrology Huashan Hospital Fudan University Shanghai China; ^2^ Nursing Department Huashan Hospital Fudan University Shanghai China; ^3^ Department of Urology Beijing Friendship Hospital Capital Medical University Beijing China; ^4^ Shanghai Topbiox Co. Ltd Shanghai China; ^5^ Shanghai Geriatric Institute of Chinese Medicine Shanghai University of Traditional Chinese Medicine Shanghai China; ^6^ Department of Pathology Yale University School of Medicine New Haven Connecticut; ^7^ Department of Biochemistry College of Basic Medicine Shanghai University of Traditional Chinese Medicine Shanghai China

**Keywords:** 5‐hydroxymethylation, cell cyclin‐dependent kinases, fisetin, renal cancer stem cells

## Abstract

As a natural flavonol, fisetin has significant inhibitory effects on many cancers. Although fisetin can inhibit kidney cancer, its effects on kidney renal stem cells (HuRCSCs) remain unknown. Our study found that renal cancer tissues and CD44+/CD105+ HuRCSCs both show high TET1 protein expression. Both in vivo and in vitro experiments showed that fisetin can effectively inhibit HuRCSC cell division and proliferation, invasion, in vivo tumourigenesis and angiogenesis. Our findings showed that fisetin can significantly decrease TET1 expression levels in HuRCSCs and overall 5hmC levels in the genomes of these cells. At the same time, ChIP‐PCR results showed that fisetin can effectively inhibit 5hmC modification levels at the CpG islands in cyclin Y (CCNY) and CDK16 and reduce their transcription and activity. Thus, we conclude that fisetin inhibits the epigenetic mechanism in renal cancer stem cells, that is, fisetin inhibits TET1 expression and reduces 5hmC modification in specific loci in the promoters of CCNY/CDK16 in HuRSCs. This in turn inhibits transcription of these genes, causing cell cycle arrest and ultimately inhibiting renal cancer stem cell activity.

## 1 INTRODUCTION

Renal cell carcinoma (RCC) is the most common primary renal parenchymal malignancy in adults. RCC originates in the epithelial cells in the proximal convoluted tubule and its incidence is second only to bladder cancer among urogenital cancers, accounting for 2%‐3% of human tumours, and 80%‐90% of malignant tumours of the kidney.^1^, ^2^, ^3^, ^4^, ^5^ RCC has high malignancy and poor prognosis, which severely threatens human health.^2^, ^3^, ^4^, ^5^ The appearance of RCC is due to a series of clinical malignant transformations in kidneys at the pathological and molecular levels.^1^, ^2^, ^3^, ^4^, ^5^ Clear cell carcinoma and papillary carcinoma (Types 1 and 2) account for most types of RCCs.^1^, ^2^, ^3^, ^4^, ^5^ Recent studies have shown that the occurrence of RCC is intimately associated with aberrant activation of many signal transduction pathways and aberrant gene methylation.^1^, ^2^, ^3^, ^4^, ^5^

DNA methylation refers to the epigenetic modification in which a methyl group is added at the cytosine (C) of DNA to form methylcytosine.^6^, ^7^ DNA methylation is intimately associated with cancer occurrence and development.^6^, ^7^ Changes in DNA methylation include hypermethylation and hypomethylation.^6^, ^7^ Generally, hypermethylation of promoter DNA indicates that the gene is silenced, whereas hypomethylation indicates that the gene is activated.^6^, ^7^ As an important epigenetic modification, DNA methylation participates widely in the regulation of gene expression, histone modification and other processes.^6^, ^7^ In many in vivo and in vitro reprogramming processes, removal of DNA methylation is extremely important in the activation of totipotent genes and reconstruction of histone modifications.^6^, ^7^ The ten‐eleven translocation (TET) protein family contains three members, namely Tet1, Tet2 and Tet3.^8^, ^9^, ^10^ These three proteins contain a conserved C‐terminal catalytic domain and classical characteristics of 2‐oxoglutarate‐ and Fe (II)‐dependent dioxygenases.^8^, ^9^ The dioxygenases in the TET family can gradually oxidize 5‐methylcytosine (5mC) in mammalian genomes to 5‐hydroxymethylcytosine (5hmC), 5‐formylcytosine (5fC) and 5‐carboxylcytosine (5caC).^6^, ^7^, ^9^, ^10^ Thymine‐DNA glycosylase (TDG) in mammals is responsible for recognition and excision of 5fC and 5caC. TDG carries out base excision repair to restore them to normal cytosine.^6^, ^7^ Recent studies showed that TET2 mutations are present in around 6% of patients with RCC. Compared with normal tissues, 5mC accumulation occurs in TET‐mutated tumours.^11^ By profiling genome 5mC and 5hmC levels in genomes, in tissues using single nucleotide resolution, researchers have proved that some 5mC is present in the genome of renal cancer tissues compared with normal tissues. However, there is significant 5hmC loss in renal cancer tissues.^11^ At the same time, studies have shown that 5hmC level in renal cancer tissues is an independent prognostic marker for renal cancer. 5hmC levels are intimately associated with overall survival.^11^

Cell division requires joint regulation by cyclin‐dependent kinases (CDKs) and cyclins. The CDK family includes 20 protein kinases that regulate cell division and proliferation. Cyclin Y (CCNY) is one of the protein members that regulate cell cycle progression.^12^, ^13^ CCNY family proteins can enhance the activity of the Wnt/β‐catenin signalling pathway in mitosis.^12^, ^14^, ^15^, ^16^, ^17^, ^18^ Zeng et al reported that CCNY‐like 1 (Ccnyl1) and CCNY are essential for embryonic development and division of progenitor cells in mice.^14^ Among the members of the cyclin superfamily, CCNY is highly conserved among cyclins and is a substrate that is regulated by CDK14 and CDK16.^12^, ^14^, ^15^, ^16^, ^17^, ^18^ CCNY positively regulates the proliferation and division of tumour cells. Down‐regulation of CCNY expression will significantly decrease the growth and division speed of tumour cells.^12^, ^14^, ^15^, ^16^, ^17^, ^18^

Fisetin (3,7,3′,4′‐tetrahydroxyflavone) is a natural flavonol that is abundant in many fruits and vegetables and has classical anti‐oxidant and anti‐tumour properties.^19^, ^20^, ^21^, ^22^, ^23^ Fisetin exhibits significant affinity with microtubule proteins and can bind to microtubules, which stabilize its structure and function.^19^, ^20^ Its binding characteristics are far superior to paclitaxel.^19^, ^20^ Fisetin inhibits the binding of Nudc and the microtubule transporter dynein/dynactin complex (a protein that regulates microtubule dynamics in cells) to significantly inhibit the division of tumour cells.^19^, ^20^, ^21^, ^22^, ^23^ In addition, fisetin up‐regulates the expression of microtubule‐associated protein 2/4 in prostate cancer to inhibit proliferation and invasion by cancer cells.^19^, ^20^, ^21^, ^22^, ^23^

In view of the aforementioned evidence, we attempt to isolate renal cancer stem cells (HuRCSCs) from the tissue samples of renal cancer patients. We attempt to prove in vivo and in vitro that fisetin regulates TET protein expression to regulate the 5‐hydroxymethylcytosine modification of genomes in HuRCSCs. We also inhibited the 5‐hydroxymethylcytosine modification at the promoter regions of CCNY/CDK16 to achieve in vivo and in vitro HuRCSC proliferation and invasion.

## 2 MATERIALS AND METHODS

### 2.1 Information on patient tissue samples

The 50 renal cancer tissue samples were collected from patients who underwent surgery for renal cancer from the Urology Departments of the Beijing Friendship Hospital affiliated to the Capital University of Medical Sciences and the Huashan Hospital affiliated to Fudan University from January 2016 to April 2018. All operations conform to ethical standards. The mean age of these 50 patients was 56 years (46‐68 years). Among them, 20 had clear cell RCC, 20 had papillary RCC, five had chromophobe RCC and five had other renal cancers.

### 2.2 Isolation and culture of stem cells

In brief,^1^, ^24^ the clear cell RCC tissues from three patients were first digested using trypsin (containing 0.02% EDTA‐Na) at 37°C for 30 minutes before the reaction was terminated using cell culture medium containing 15% foetal bovine serum. The volume of the cell suspension was adjusted and 4 μL FITC‐labelled rabbit anti‐human CD44 monoclonal antibody and Cy3‐labelled rabbit anti‐human CD105 antibody (eBioscience) were added to 100 μL of cell suspension and incubated in the dark at 4°C for 30 minutes. Pre‐cooled PBS was used to readjust the volume of the cell suspension to 500 μL. A flow cytometer (BD FACSAria) was used to select CD44+/CD105+ HuRCSCs and CD44‐/CD105‐ normal human renal cancer cells, HuNRCCs. All cells were resuspended in complete cancer stem cell culture medium: DMEM:F12 (HyClone), supplemented with 10 ng/mL basic fibroblast growth factor, 10 ng/mL epidermal growth factor, 5 μg/mL insulin, 1% bovine serum albumin (BSA) and 5% knockout serum replacement (KnockOut SR) (all in Gibco).

### 2.3 HE staining

In brief,^1^ 4 μ thick sections were cut from paraffin blocks and pasted to slides. Xylene was used for clearing and unequal concentrations of ethanol were used for dehydration. Haematoxylin was added and the slides were stained for 5 minutes before differentiation using hydrochloric acid in ethanol. This is followed by bluing using ammonium solution, followed by washing with distilled water for 10 minutes. Following that, eosin was added and the slides were stained for 2 minutes before washing with distilled water for 10 minutes. Ethanol dehydration was carried out, followed by xylene clearing for 2 minutes, and sealing using neutral resin.

### 2.4 Immunofluorescence staining

In brief, 4% paraformaldehyde was added to cell or tissue sections for fixing at room temperature for 40 minutes, followed by antigen retrieval, after which blocking solution (PBS containing 0.5% Triton X‐100 and 5% goat serum) was added for blocking at 37°C for 40 minutes. Primary antibodies (Table S1) were then added and allowed to react at 37°C for 40 minutes. The wash solution (PBS containing 0.5% Triton X‐100) was used to wash the sections thrice at room temperature, for 5 minutes each time. Secondary antibodies (Table S1) were then added and allowed to react at 37° for 40 minutes. The wash solution (PBS containing 0.5% Triton X‐100) was used to wash the sections thrice at room temperature, for 5 minutes each time. Small amounts of 4,6‐diamidino‐2‐phenylindole (DAPI) were added as a mounting medium.

### 2.5 Annexin IV/PI staining and flow cytometry detection

Experiments were carried out using the Annexin V‐FITC Apoptosis Detection Kit (Beyotime Biotechnology, China) according to the manufacturer's instructions. In brief, trypsin was used to digest adherent cells into single cells before PBS was used to wash the cells Centrifugation was used to remove residual solution and 195 μL Annexin V‐FITC binding solution was used to gently resuspend the cells. Subsequently, 5 μL Annexin V‐FITC was added before 10 μL propidium iodide staining solution was added and the solution was gently mixed. The solution was incubated in the dark at 20°C for 30 minutes before a flow cytometer (Cytomics FC 500; BECKMAN) was used for detection.

#### 2.5.1 Methyl thiazolyl tetrazolium assay

In brief,^1^ the number of cells in various groups was adjusted and 2 × 10^3^/mL of cells was subcultured onto a 96‐well cell culture plate. On the second day, 20 μL of methyl thiazolyl tetrazolium (MTT; Sigma‐Aldrich Chemical) solution was added to various groups and incubated at 37°C for 3 hours. The culture medium was discarded and 100 μL DMSO was added to dissolve formazan. The cell culture plates were placed in a microplate reader and the absorbance at 570 nm was recorded. The calculation formula for inhibition rate of cell proliferation (%) is: (1‐OD value of cells in the experiment group/OD of cells in the control group) × 100%.

#### 2.5.2 PI stain and FCM assay

In brief,^1^ 5 × 10^5^/mL of cells was collected and 1 mL of 70% pre‐cooled ethanol was added for 48 hours of fixation. The cells were centrifuged at 252 *g* for 5 minutes at 4°C. The cell pellet was collected and PI staining solution (Sigma Chemicals) was added and the cells were incubated in the dark at 4°C for 30 minutes. A flow cytometer (Cytomics FC 500; BECKMAN) was used to analyse the cell cycle distribution of various groups of cells. The CellQuest software was used for data analysis.

#### 2.5.3 Transwell migration assay

In brief,^1^ 200 μL of serum‐free cell culture medium containing 2 × 10^3^/mL cells was inoculated into the upper chambers of 8.0 μm/well Transwell chambers. Six hundred‐microlitre complete culture medium containing 10% FBS was added to the lower chambers of Transwell chambers. The cells were cultured at 37°C, 5% CO_2_ conditions for 48 hours. Cells that had adhered to the membrane surface were fixed using 4% paraformaldehyde at room temperature for 30 minutes and stained with DAPI (Sigma‐Aldrich Chemical) for 10 minutes. Three non‐overlapping visual fields were selected for counting of cell numbers under the microscope.

#### 2.5.4 In vivo xenograft experiments

In brief,^1^ 1 × 10^5^/mL of cells at the logarithmic growth phase was subcutaneously inoculated in BALB/C^nu/nu^ mice. Each group contains three mice (6‐8 week‐old male BALB/C^nu/nu^ mice were provided by the Experimental Animals Centre of Fudan University). After observing the mice for 64 days, the mice were killed and tumours were extracted. The tumours were weighed and tumour volume was calculated using the following formula: Tumour volume (mm3) = (ab2)/2 (a: the longest axis (mm), b: the shortest axis (mm)).

### 2.6 RNA extraction and analysis by quantitative real‐time PCR

The Trizol Reagent (Invitrogen) was used to extract total RNA from cells from various groups, according to the manufacturer's instructions. After DNAse I (Sigma‐Aldrich) treatment, total RNA was quantitated and reverse transcription was carried out using the ReverTra Ace‐α First Strand cDNA Synthesis Kit (TOYOBO) to synthesize cDNA. Quantitative real‐time (qRT)‐PCR was carried out using the RealPlex4 real‐time PCR detection system from Eppendorf Co. Ltd (Germany). The SyBR Green RealTime PCR Master MIX (TOYOBO) was used as a fluorescent dye for nucleic acid amplification. A total of 40 amplification cycles were carried out for qRT‐PCR: denaturation at 95°C for 15 seconds, annealing at 58°C for 30 seconds and extension at 72°C for 42 seconds. The 2‐ΔΔCt method was used to calculate the relative expression of genes, where ΔCt = Ct_genes − Ct_18sRNA; ΔΔCt = ΔCt_all_groups − ΔCt_blankcontrol_group. The mRNA expression levels were corrected using the 18s rRNA expression levels. The primers required for the amplification of each gene were described in previous studies.^1^, ^9^, ^12^, ^14^, ^15^

#### 2.6.1 Western blot

In brief,^1^ the total proteins from cells from various groups were run on a 12% denaturing SDS‐PAGE gel. After electrophoresis was completed, the proteins were transferred into PVDF membranes (Millipore). After blocking and washing, the membranes were incubated with primary antibodies at 37°C for 45 minutes (Table S1). After complete washing, the membranes were incubated with secondary antibodies at 37°C for 45 minutes. The membranes were washed four times with TBST, for 14 minutes per wash. Following that, enhanced chemiluminescence (ECL) (ECL kit, Pierce Biotechnology) was used for exposure and development (Sigma‐Aldrich Chemical).

#### 2.6.2 Dot blot

In brief,^9^ total DNA was extracted from cells in various groups and the DNA concentration was adjusted to 600 ng/μL. DNA suspension (2 μL) was added to a cationic membrane before UV cross‐linking for 40 seconds, followed by drying at 80°C for 30 minutes. Following that, blocking solution (PBS containing 0.05% Tween‐20, and 5% BSA) was used for blocking at room temperature for 3 hours. Primary antibodies (Table S1) were then added and allowed to react at 4°C overnight. The wash solution (PBS containing 0.05% Tween‐20) was used to wash the membranes thrice at room temperature, for 15 minutes each time. HRP‐labelled secondary antibodies (Table S1) were then added and allowed to react at 37°C for 40 minutes. The membranes were washed thrice at room temperature, for 15 minutes each time. Following that, ECL (ECL kit; Pierce Biotechnology) was used for exposure and development.

## 3 RESULTS

### 3.1 TET1 is highly expressed in renal cancer tissues

**Figure 1:**
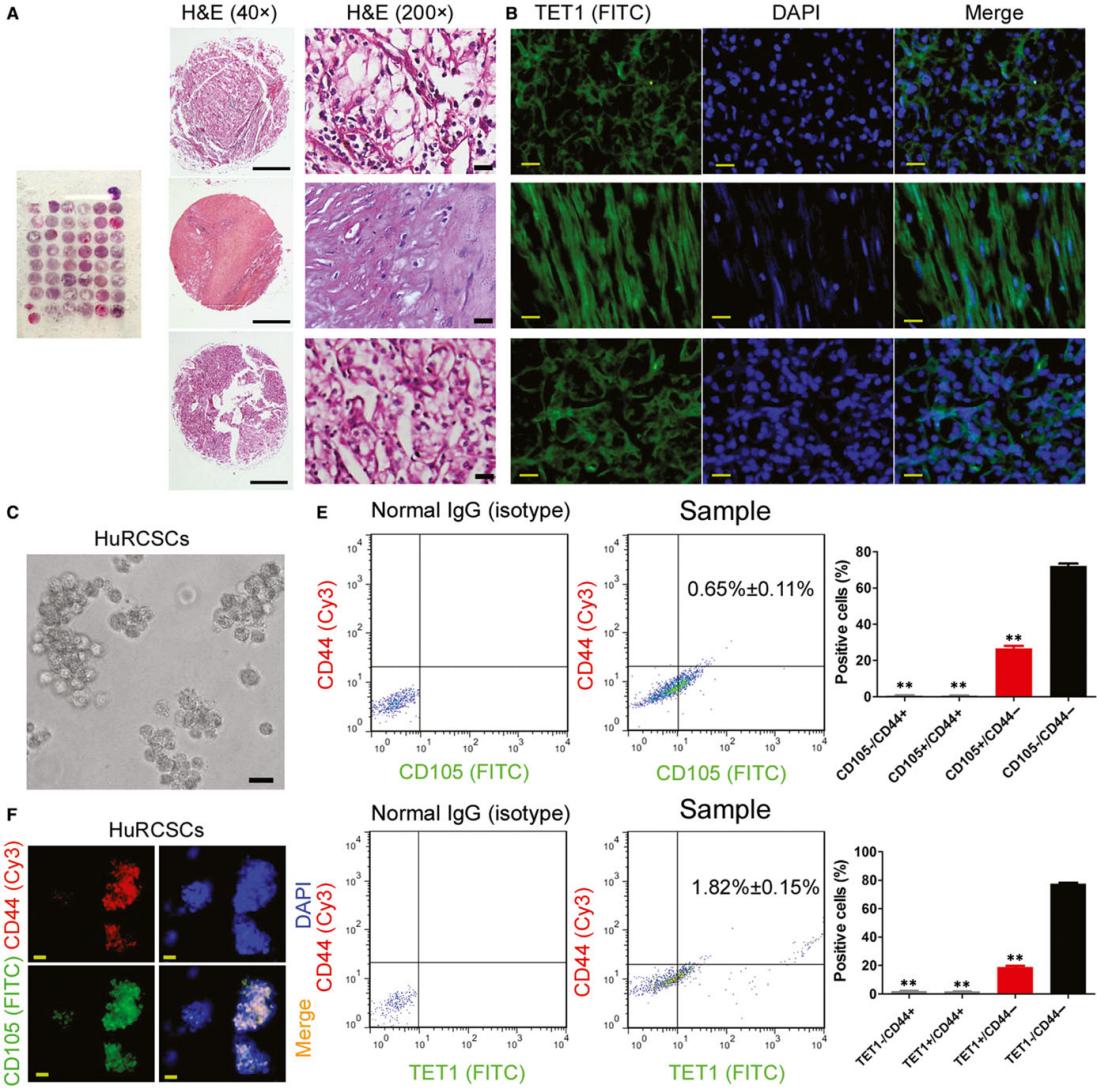
TET1 is highly expressed in renal cancer tissues. (A) HE staining and pathological identification of renal cancer microarray. Scale bar = 30 μm. (B) Immunofluorescence staining and identification of TET1 protein. Magnification: 200×. Scale bar = 30 μm. (C) Cell morphology of CD44+/CD105+ renal cancer stem cell in suspension culture Magnification: 200×. Scale bar = 30 μm. (D) Immunofluorescence staining and identification of co‐expression of CD44 and CD105 proteins on HuRCSCs. Magnification: 200×. Scale bar = 30 μm. (E) Flow cytometry determination of the proportion of renal cancer cells with co‐expression of CD44, CD105, and TET1. **P < 0.01 vs CD105‐/CD44‐ group; n = 3; *t* test

We collected renal cancer tissue samples from 50 patients to generate a tissue microarray (Figure 1A). Through HE staining, we selected three tissues from the tissue microarray, which represent clear cell RCC, papillary RCC and chromophobe RCC as study cases (Figure 1A). Immunofluorescence staining results showed that TET1 protein is highly expressed, regardless of the type of renal cancer tissue (Figure 1B). Through flow cytometry, we obtained the CD44+/CD105+ renal cancer stem cell (HuRCSCs) subpopulation. This subpopulation accounted for an extremely low proportion of the entire renal cancer tissue (around 0.65 ± 0.11%) (Figure 1C‐E). In addition, FCM results showed that renal cancer cells co‐expressing TET2 and CD44 accounted for 1.82 ± 0.15% of the entire cell population. Immunofluorescence staining and identification showed that the HuRCSCs obtained from screening have a high expression of the CD44 and CD105 markers (Figure 1D and E, Figure S1). It can be seen that TET1 expression is positively correlated with the degree of malignancy of renal cancer and is highly expressed in HuRCSCs.

### 3.2 Fisetin inhibits in vivo and in vitro activity of HuRCSCs

**Figure 2:**
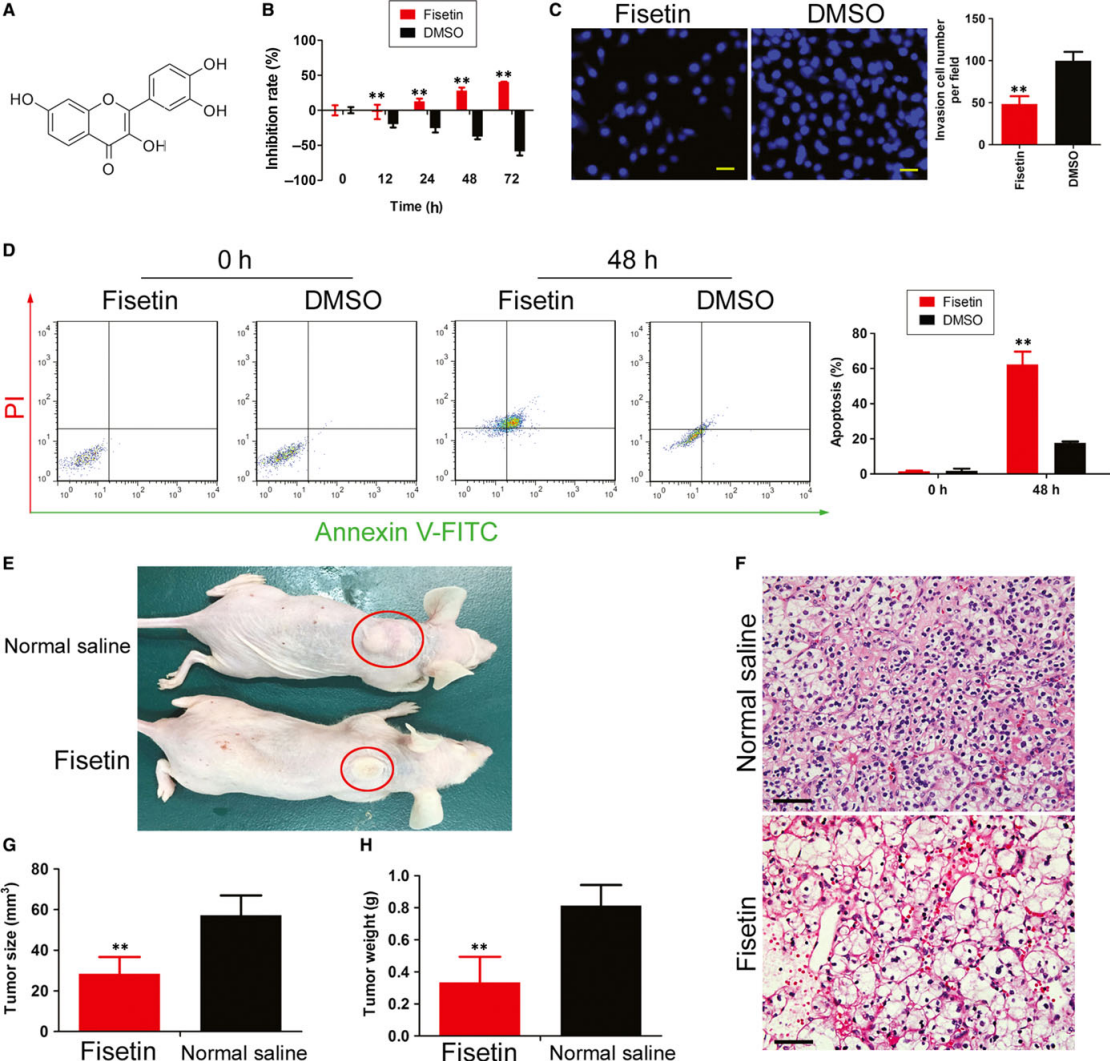
Fisetin inhibits in vivo and in vitro activity of HuRCSCs. (A) Molecular structure of fisetin. (B) MTT assay results showed that fisetin significantly inhibits the in vitro proliferation of HuRCSCs. **P < 0.01 vs DMSOgroup; n = 3; *t* test. (C) Transwell experiment results showed that fisetin decreases the invasiveness of HuRCSCs on extracellular matrix. **P < 0.01 vs DMSOgroup; n = 3; *t* test. Scale bar = 30 μm. (D) Annexin V/PI staining and FCM test results showed that fisetin can significantly increase the proportion of apoptotic cells in HuRCSCs. **P < 0.01 vs DMSOgroup; n = 3; *t* test. (E) Tumour‐bearing mouse model (F) HE staining shows that the type of tumour was clear cell renal cell carcinoma. Magnification: 200×. Scale bar = 30 μm. (G) The volume of tumours formed in the fisetin treatment group was significantly smaller than those formed from the control group. **P < 0.01 vs physiological saline group; n = 3; *t* test. (H) The weight of tumours formed in the fisetin treatment group was significantly smaller than those formed from the control group. **P < 0.01 vs physiological saline group; n = 3; *t* test

**Figure 3:**
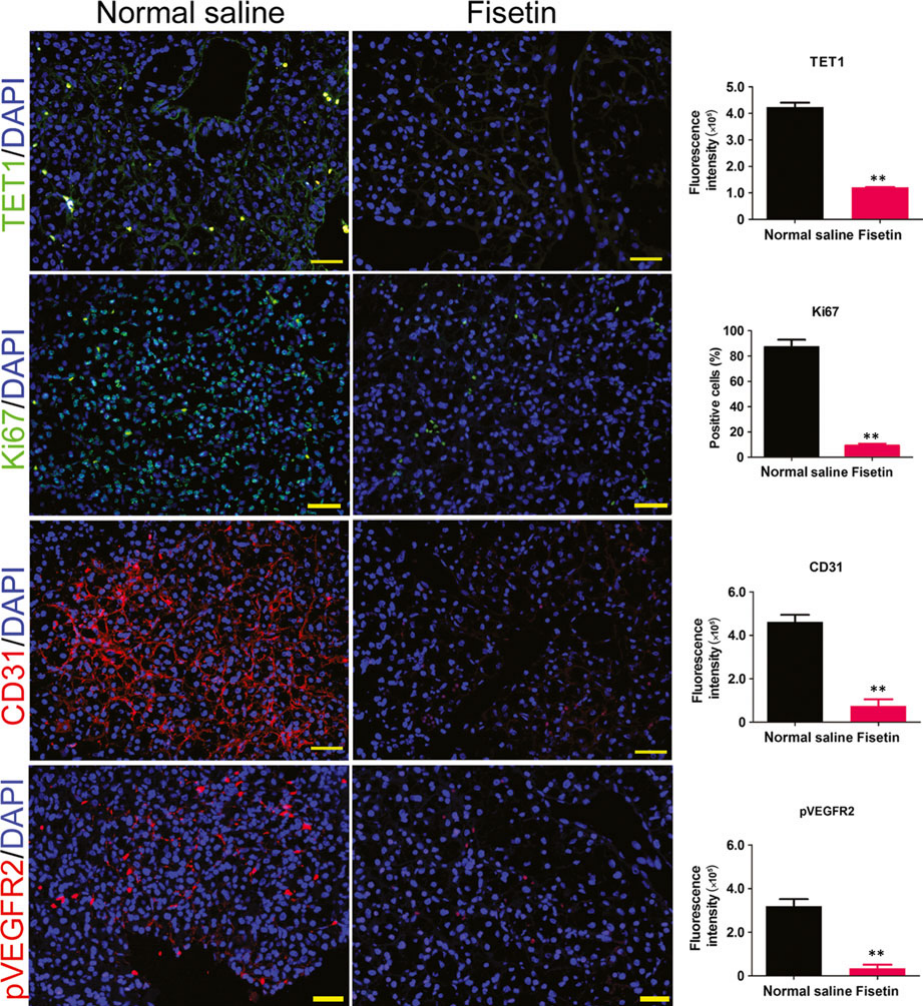
Fisetin inhibits in vivo proliferation and angiogenesis in HuRCSCs. Magnification: 200×. **P < 0.01 vs physiological saline group; n = 3; *t* test. Scale bar = 30 μm

According to the results of previous studies, we selected the recommended IC50 concentration (50 μmol/L) to treat HuRCSCs.^19^, ^20^ MTT assay results showed that cell proliferation was inhibited after fisetin was co‐cultured with HuRCSCs for 12 hours. As co‐culture duration increases, inhibition of cell proliferation significantly increased. Compared with the control group (DMSO), this difference was statistically significant (Figure 2A and B). The Transwell experiment results showed that co‐culture of fisetin and HuRCSCs for 48 hours can significantly decrease the invasiveness of HuRCSCs on the extracellular matrix (Figure 2C). At the same time, Annexin V/PI staining and FCM test results showed that fisetin can significantly increase the proportion of apoptotic cells in HuRCSCs after fisetin was co‐cultured with HuRCSCs for 48 hours (Figure 2D). In addition, equal amounts of HuRCSCs were inoculated subcutaneously at the scapular of NOD mice. The mice were randomized into two groups, of which intraperitoneal injection of fisetin was carried out in one group whereas intraperitoneal injection of an equal volume of physiological saline was carried out in the other group.^19^, ^20^ Tumours could be observed on the backs of the mice in the physiological saline treatment group on Day 32, whereas no tumours were found on the backs of the fisetin‐treated nude mice group. Mice were killed on Day 64 and tumours on the backs were collected for the measurement of tumour volume and weight. Results showed that the tumours from the fisetin treatment group were significantly smaller than those formed from the control group in terms of both tumour volume and weight (Figure 2E, G and H). However, HE staining shows that the type of tumour formed in the two groups was clear cell RCC (Figure 2F). However, immunofluorescence staining results showed that the tumours from the fisetin‐treated group have significantly reduced Ki67 positive cell numbers, 5hmC oxygenase TET1 and biomarkers for tumour angiogenesis, CD31 and pVEGFR2 compared with the control group (Figure 3). In vivo and in vitro experiments showed that fisetin significantly inhibits HuRCSC proliferation, invasion, in vivo tumourigenesis and angiogenesis.

### 3.3 Fisetin inhibits TET1 expression and genomic 5hmC levels in HuRCSCs

**Figure 4:**
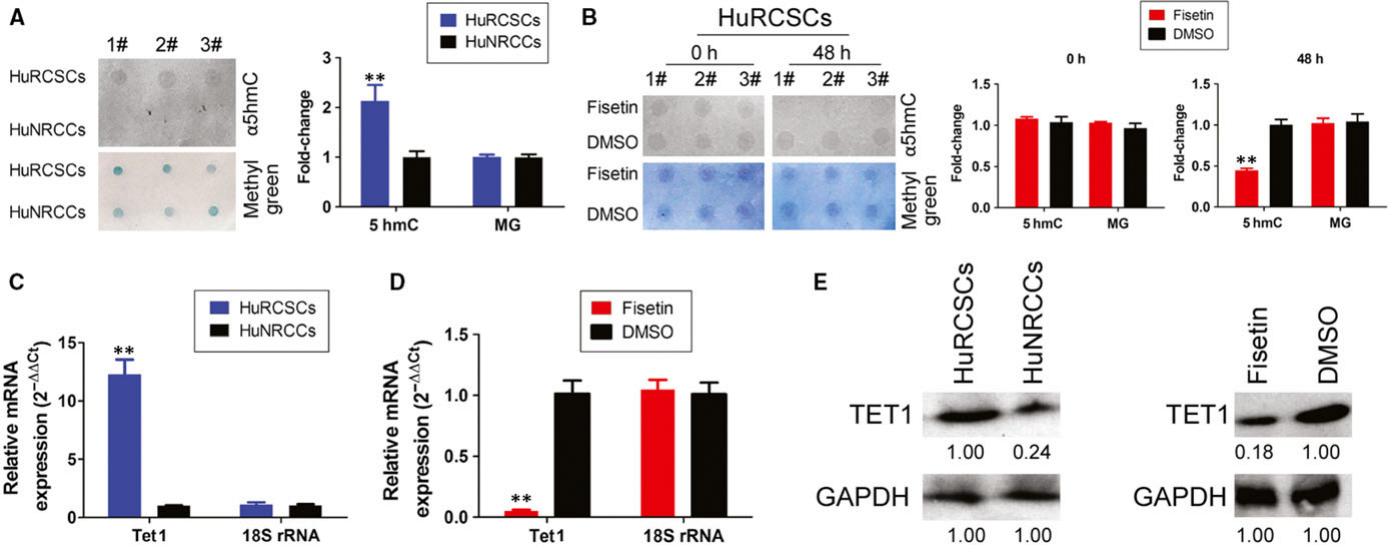
Fisetin inhibits TET1 expression and 5hmC modification levels in the genomes of HuRCSCs. (A) Dot blot results showed that HuRCSCs have a significantly higher genomic 5hmC level than HuNRCCs. **P < 0.01 vs HuNRCCs; n = 3; *t* test. (B) The genome 5hmC levels of the fisetin treatment group were significantly lower than cells from the control group. **P < 0.01 vs DMSO group; n = 3; *t* test. (C) qPCR results showed that HuRCSCs have significantly higher Tet1 mRNA levels than HuNRCCs. **P < 0.01 vs HuNRCCs; n = 3; *t* test. (D) qPCR results showed that fisetin significantly decreases endogenous Tet1 mRNA levels in HuRCSCs. **P < 0.01 vs DMSO group; n = 3; *t* test. (E) Western blotting results showed that HuRCSCs have a significantly higher expression of TET1 protein than HuNRCCs. However, fisetin significantly inhibits the expression of endogenous TET1 in HuRCSCs

Firstly, dot blot was used to determine genomic 5hmC levels in HuRCSCs. The experiment results showed that genomic 5hmC levels in CD44+/CD105+ HuRCSCs were significantly higher than those in CD44‐/CD105‐ HuNRCCs (Figure 4A). However, the genomic 5hmC levels of the HuRCSCs that were pre‐treated with fisetin for 48 hours were significantly lower than those of cells from the control group (pre‐treatment with an equal volume of DMSO) (Figure 4B). In addition, qPCR and Western blot results showed that HuRCSCs have a significantly higher Tet1 mRNA and protein expression than HuNRCCs (Figure 4C and E). However, when HuRCSCs were pre‐treated with fisetin for 48 hours, the expression levels of endogenous TET1 were significantly lower than cells from the control group (Figure 4D and E). The experiment results showed that fisetin can effectively inhibit TET1 expression in HuRCSCs and reduce overall genomic 5hmC levels in HuRCSCs.

### 3.4 Fisetin inhibits TET1‐induced CCNY/CDK16 activation

**Figure 5:**
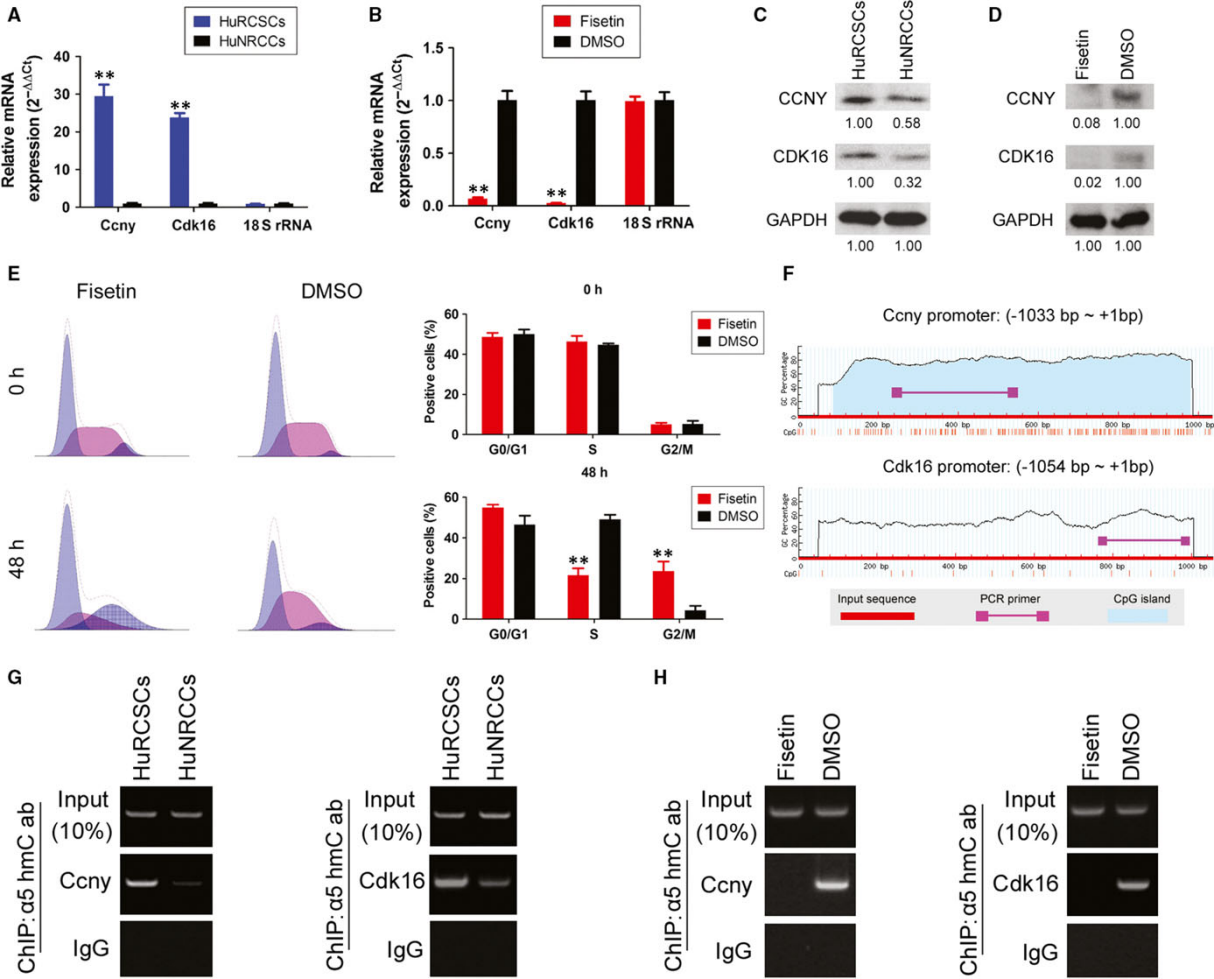
Fisetin inhibits TET1‐induced CCNY/CDK16 activation. (A) qPCR results showed that HuRCSCs have significantly higher Ccny and Cdk16 mRNA levels than HuNRCCs. **P < 0.01 vs HuNRCCs; n = 3; *t* test. (B) qPCR results showed that fisetin significantly decreases endogenous Ccny and Cdk16 mRNA levels in HuRCSCs. **P < 0.01 vs DMSO group; n = 3; *t* test. (C) Western blotting results showed that HuRCSCs have a significantly higher expression of CCNY and CDK16 protein than HuNRCCs. (D) Western blotting results showed that fisetin significantly inhibits the expression of endogenous CCNY and CDK16 in HuRCSCs. (E) Cell cycle FCM results shows that cell cycle progression was significantly blocked after HuRCSCs were treated with fisetin for 48 h. In addition, the proportion of cells in the S phase was significantly decreased while the proportion of cells in the G2/M phase was significantly increased. **P < 0.01 vs DMSO group; n = 3; *t* test. (F) CpG island prediction results for specific regions in the promoters of the Ccny and Cdk16 genes and ChIP‐PCR primer coverage loci. (G) 5hmC ChIP‐PCR results showed that among the complexes that cross‐link with the anti‐5hmC antibody, positive bands for specific promoter regions in the Ccny and Cdk16 genes can be obtained by PCR. (H) 5hmC ChIP‐PCR results showed that post‐fisetin treatment of HuRCSCs, among the complexes that cross‐link with the anti‐5hmC antibody, almost no positive bands for specific promoter regions in the Ccny and Cdk16 genes can be obtained by PCR

Firstly, qPCR and Western blot results showed that HuRCSCs have significantly higher Ccny and Cdk16 mRNA and protein expression than HuNRCCs (Figure 5A and C). However, when HuRCSCs were treated with fisetin for 48 hours, the expression levels of endogenous CCNY and CDK16 were significantly lower than that of cells from the control group (Figure 5B and D). Cell cycle FCM results also show that cell cycle progression was significantly blocked after HuRCSCs were treated with fisetin for 48 hours. In addition, the proportion of cells in the S phase significantly decreased, whereas the proportion of cells in the G2/M phase significantly increased, compared with the control group (Figure 5E). We designed primers for specific regions in the promoters of the Ccny and Cdk16 genes by using bioinformatics tools (http://www.urogene.org/cgi-bin/methprimer/methprimer.cgi) (Figure 5F). The 5hmC ChIP‐PCR experiment results showed that among the complexes that cross‐link with the anti‐5hmC antibody, positive bands for specific promoter regions in the Ccny and Cdk16 genes can be obtained by PCR (Figure 5G, Figure S2). However, after fisetin treatment of HuRCSCs, almost no positive bands for specific promoter regions in the Ccny and Cdk16 genes can be obtained by PCR (Figure 5H, Figure S2). Therefore, the experiment results showed that fisetin can inhibit endogenous TET1 activity in HuRCSCs to reduce 5hmC modification in the specific promoter regions in the Ccny and Cdk16 genes and decrease CCNY/CDK16 protein activity.

## 4 DISCUSSION

Currently, a larger number of studies have pointed out that a special cell subpopulation exists in tumour tissues, that is, cancer stem cells. Cancer stem cells have high proliferation and invasion abilities, show significant resistance to chemotherapy agents and can easily induce tumour metastasis and relapse. Therefore, they are one of the direct factors for poor prognosis in cancer patients. In vitro and in vivo experiments pointed out that the drug resistance of cancer stem cells is greater than that of ordinary cancer cells. In addition, cancer stem cells are resistant to a broader range of chemotherapy drugs, such as platinum‐based drugs, paclitaxel, gemcitabine, etc. Inhibiting drug resistance in cancer stem cells is a key to preventing reactivation of tumour cells and tumour recurrence. As conventional chemotherapy agents have limited cytotoxicity towards cancer stem cells, more researchers are testing out natural products in the hopes of identifying substances than can inhibit cancer stem cells. As research intensifies, awareness on the anti‐cancer properties of flavonoids is increasing. Flavonoids refer to a series of compounds that contain two phenolic hydroxyl benzene rings (A and B rings) that are linked by three central carbon atoms. The basic skeleton is 2‐phenylchromone. Natural flavonoids include flavones, flavonol, flavonones, flavanonol, anthocyanidins, flavan‐3,4‐diols, xanthones, chalcones and biflavonoids, for a total of 15 types. In addition, the structures of some flavonoids are complex, which include ficine, isoficine and other flavonoid alkaloids. Vitamin P, tea polyphenols, gingko leaf flavonoids, propolis, quercetin, curcumin, purerarin, ginkgetin and fisetin are all flavonoids with which the public is familiar. Flavonoids have classical functions as an anti‐oxidant, in anti‐ageing, in maintenance of cardiovascular system and in anti‐inflammation. In addition, more studies have found that Vitamin P, tea polyphenols, propolis, apigenin, curcumin, etc., have significant effects in inhibiting tumour proliferation and invasion. At the same time, our previous study found that curcumin can regulate microRNA expression to inhibit the in vivo and in vitro proliferation and invasion of prostate cancer stem cells. Therefore, the above study results provided a scientific basis for the anti‐cancer hypothesis of flavonoids. Fisetin is a flavonoid derived from fruits and vegetables. Reports have shown that fisetin can inhibit in vivo and in vitro proliferation and tumourigenicity of different tumours. However, there are currently only a few reports that show that fisetin has some inhibitory effects on cancer stem cells, particularly renal cancer stem cells.

In the present study, we investigated two questions: First, does fisetin have significant inhibitory effects on the in vivo and in vitro proliferation, invasion, tumourigenicity of renal cancer stem cells and tumour angiogenesis? Second, if fisetin exhibits inhibitory effects on renal cancer stem cells, what are the effector mechanisms involved? To answer these questions, we first isolated and cultured human renal cancer stem cells (CD44+/CD105+). Through fisetin treatment, we found that fisetin has significant inhibitory effects on in vivo and in vitro proliferation, invasion, tumourigenicity of renal cancer stem cells and tumour angiogenesis. The experiment results showed that fisetin can inhibit renal cancer stem cells. Following that, we further analysed the effector mechanisms by which fisetin inhibits cancer stem cells. Firstly, we discovered an interesting phenomenon: all pathological sections from different types of renal cancer tissues from patients showed high TET1 protein expression. At the same time, we found that CD44+/CD105+ renal cancer cells have significantly higher expression of TET1 than CD44‐/CD105‐ renal cancer cells. However, endogenous TET1 expression was significantly decreased after renal cancer stem cells were treated with fisetin and this decrease is directly proportional to treatment duration. Therefore, the experiment results showed that TET1 may be one of the important targets of fisetin. On the other hand, flow cytometry showed that fisetin‐treated renal cancer stem cells showed significant cell cycle arrest. This suggests that fisetin inhibits one or some CDKs and cyclins to cause renal cancer stem cells to be unable to successfully divide. Therefore, we focus our attention on CCNY and CDK16. Through molecular biology experiments, we found that the mRNA and protein expression levels of endogenous CCNY and CDK16 were significantly decreased in fisetin‐treated renal cancer stem cells. Therefore, we suggest that fisetin inhibition of mitosis in renal cancer stem cells is achieved through inhibition of CCNY and CDK16 expression. Considering that fisetin can inhibit TET1 expression, we suggest an intrinsic relationship between fisetin, TET1 and CCNY/CDK16.

**Figure 6:**
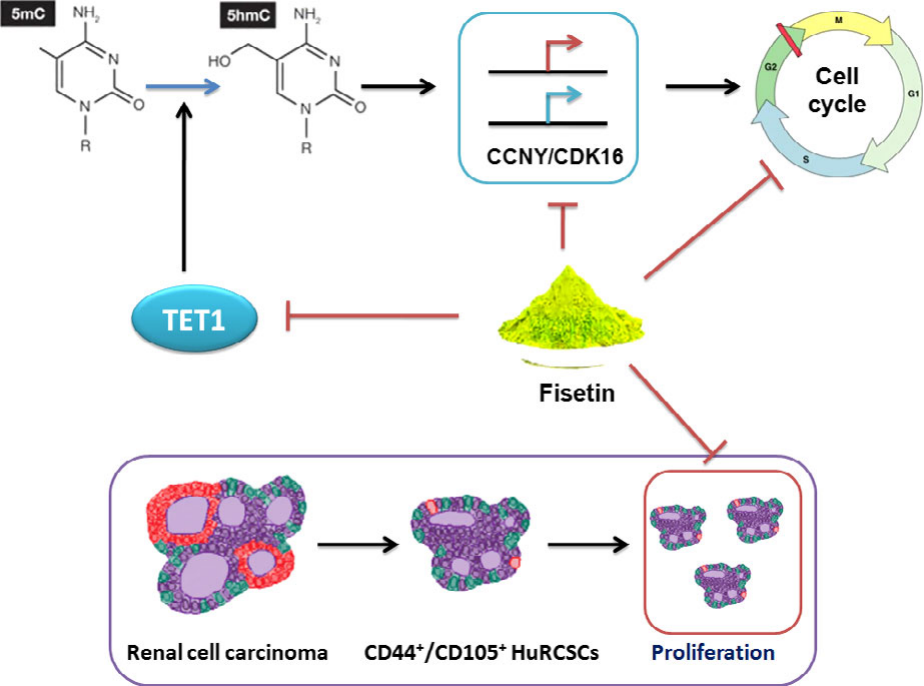
Fisetin inhibits TET1 activity and CCNY/CDK16 promoter methylation levels to inhibit the proliferation and invasion of renal cancer stem cells

Ten‐eleven translocation protein family members can catalyse genomic DNA 5mC hydroxylation. The discovery of the biological function of TET proteins provides a new understanding of 5mC demethylation mechanisms.^25^, ^26^, ^27^ This is because 5hmC may be an important intermediate in 5mC demethylation. 5mC can be further catalysed by activation‐induced cytidine deaminase (AID) into 5‐hydroxymethyluracil (5hmU). 5hmU can be recognized and excised by TDG^10^ before base excision repair is carried out to convert that locus into cytosine, thereby achieving DNA demethylation.^25^, ^26^, ^27^ Some studies pointed out that the TET1 protein contains a nuclear localisation sequence and can bind to DNA.^25^, ^26^, ^27^ Therefore, TET1 mainly carries out its physiological functions in the cell nucleus. At the same time, TET1 is highly expressed mainly in foetal tissues and stem cells.^25^, ^26^, ^27^ Based on the aforementioned clues, we analysed the 5hmC modification status on CpG islands in the promoter regions of CCNY and CDK16. The experiment results showed that the overall 5hmC modification levels of genomic DNA in fisetin‐treated renal cancer stem cells were significantly reduced. At the same time, 5hmC modification on the CpG islands in the promoter regions of CCNY and CDK16 was significantly decreased. We suggested that this phenomenon is directly associated with down‐regulation of TET1 protein expression (Figure 6).

Therefore, in summary, we found that fisetin inhibits the epigenetic mechanism in renal cancer stem cells, that is, fisetin inhibits TET1 expression and reduces 5hmC modification in specific loci in the promoters of CCNY/CDK16 in renal cancer stem cells. This inhibits transcription of these genes, causing cell cycle arrest and ultimately inhibiting renal cancer stem cell division.

## CONFLICT OF INTEREST

The authors declare that they have no competing interests.
