# Supplementary material for: Fisetin decreases TET1 activity and CCNY/CDK16 promoter 5hmC levels to inhibit the proliferation and invasion of renal cancer stem cell
Source: J Cell Mol Med. 2018 Nov 8;23(2):1095–105. doi: 10.1111/jcmm.14010 (PMC6349178; doi:10.1111/jcmm.14010)
Supplement: Supplementary file 1 [file JCMM-23-1095-s001.docx]

**Fisetin decreases TET1 activity and CCNY/CDK16 promoter 5hmC levels to inhibit the proliferation and invasion of renal cancer stem cell**

Yibing Si^1,2*^, Junfeng Liu^1,2*^, Hongliang Shen^3*^, Chen Zhang^1,2^, Yuanhao Wu^1^, Yongyi Huang^4^, Zhangbin Gong^7^, Jun Xue^1#^, Te Liu^5, 6#^

^1^ Division of Nephrology, Huashan Hospital, Fudan University, Shanghai 200040, China

^2^ Nursing Department, Huashan Hospital, Fudan University, Shanghai 200040, China

^3^ Department of Urology, Beijing Friendship Hospital, Capital Medical University, Beijing 100050, China

^4^ Shanghai Topbiox Co., Ltd, Shanghai 200231, China

^5^ Shanghai Geriatric Institute of Chinese Medicine, Shanghai University of Traditional Chinese Medicine, Shanghai 200031, China

^6^ Department of Pathology, Yale University School of Medicine, Connecticut 06520, USA

^7^ Department of Biochemistry, College of Basic Medicine, Shanghai University of Traditional Chinese Medicine, Shanghai 201203, China

* These authors contributed equally to this work and shared the first authorship.

^#^ Corresponding author: Prof. Jun Xue, Division of Nephrology, Huashan Hospital, Fudan University, Shanghai, China, 200040, Phone: 86-21-528877999; Fax: 86-21-52887799; E-Mail: xuejun@fudan.edu.cn. Dr. Te Liu, Department of Pathology, Yale University School of Medicine, 10 Amistad St, New Haven, Connecticut 06520, USA, Phone: +1-203-7856047; Fax: +1-203-7852293; E-Mail: te.liu@yale.edu.


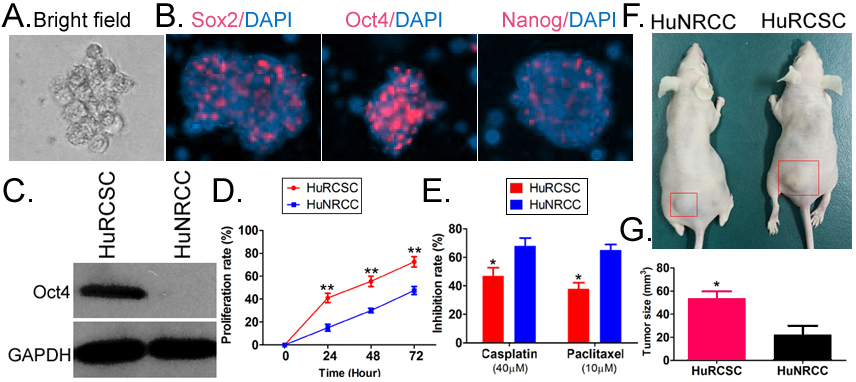


**Figure S1. Isolation and Identification of CD44+/CD1105+ HuRCSCs.** (A) The CD44+/CD1105+ HuPCaSCs form small, non-adherent, non-symmetric spheres under stem cell-selective conditions; magnification ×200. (B) Expression of stem cell markers Oct4, Nanog and Sox2 in each cells were determined by Immunofluorescence staining assay. (C) Expression levels of Oct4 in each cells were tested by western blot. The speed of cell growth in CD44+/CD105+ HuRCSCs group was faster significantly than it in CD44-/CD105- HuNRCCs group. ** p<0.01 vs CD44-/CD105- HuNRCCs; t test; n=3. (E) The inhibition rates of each cells treated with casplatin or paclitaxel were tested by MTT assay; * p<0.05 vs CD44-/CD105- HuNRCCs; t test; n=3. (F) About 1×105/ml human prostate cancer cells (CD44+/CD133+ or CD44-/CD133-) were inoculated s.c in BALB/c nude mice. (G) The tumours from the CD44+/CD105+ HuRCSCs group were significantly bigger than those formed from the CD44-/CD105- HuNRCCs group in terms of both tumour volume. * p<0.05 vs CD44-/CD105- HuNRCCs; t test; n=3.


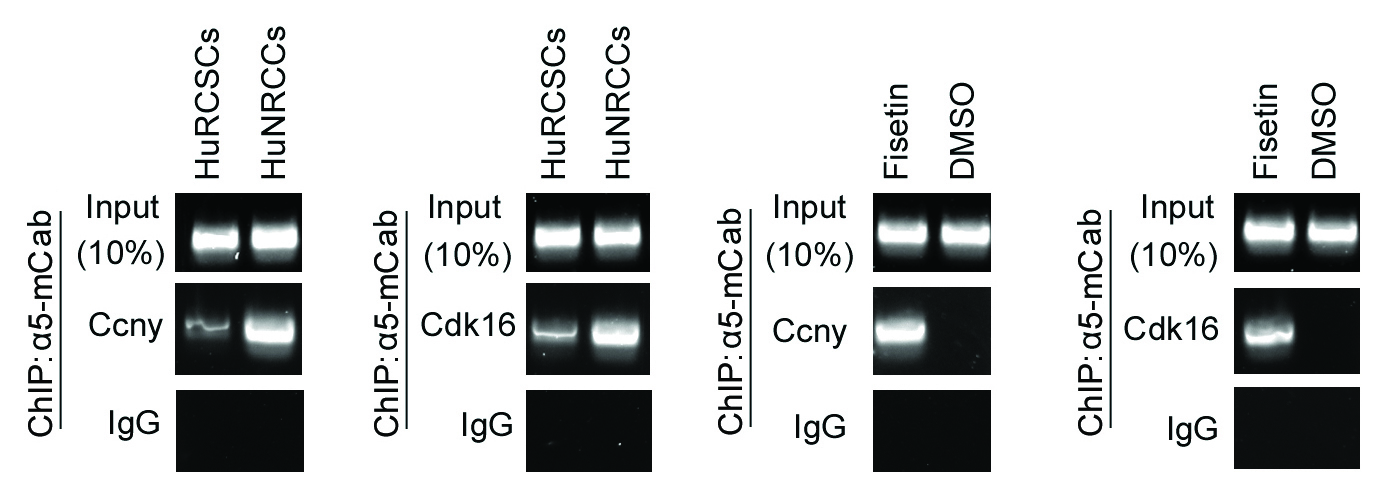


**Figure S2. The results of 5-mC ChIP-PCR assay.** 5-mC ChIP-PCR results showed that among the complexes that cross-link with the anti-5-mC antibody, positive bands for specific promoter regions in the Ccny and Cdk16 genes can be obtained by PCR in HuNRCCs. Additional, 5-mC ChIP-PCR results showed that post-fisetin treatment of HuRCSCs, among the complexes that cross-link with the anti-5-mC antibody, almost positive bands for specific promoter regions in the Ccny and Cdk16 genes can be obtained by PCR.

**Table S1 Antibodies**

| **Antibodies** | **Companies** | **Applications** |
| --- | --- | --- |
| Rabbit Anti-TET1 (ab105475) | Abcam, Inc. (MA, USA) | IF (1:100)  WB(1:1000) |
| Rabbit anti-human CD44 (#37259) | Cell Signaling Technology, Inc. (MA, USA) | IF (1:100) |
| Mouse anti-CD105 (ab11414) | Abcam, Inc. (MA, USA) | IF (1:100) |
| Rabbit anti-Ki67 (#9129) | Cell Signaling Technology, Inc. (MA, USA) | IF (1:100) |
| Rabbit anti-CD31 (#77699) | Cell Signaling Technology, Inc. (MA, USA) | IP (1:100) |
| Rabbit anti- 5-Hydroxymethylcytosine (5-hmC)( #51660) | Cell Signaling Technology, Inc. (MA, USA) | Dot blot (1:1000)  IP (1:100) |
| Rabbit anti-5-Methylcytosine (5-mC) (#28692) | Cell Signaling Technology, Inc. (MA, USA) | Dot blot (1:1000)  IP (1:100) |
| Rabbit anti-Cyclin Y (ab237677) | Abcam, Inc. (MA, USA) | WB(1:1000) |
| Rabbit anti-CDK16 (sc-53410) | Santa Cruz, Inc. (MA, USA) | WB(1:1000) |
| Rabbit anti-Phospho-VEGF Receptor 2 (Tyr1175) (#2478) | Cell Signaling Technology, Inc. (MA, USA) | IF (1:100) |
| Goat Anti-Rabbit IgG H&L (Cy3 ®) preadsorbed (ab6939) | Abcam, Inc. (MA, USA) | IF(1:100) |
| Goat Anti-Mouse IgG H&L (FITC) (ab6785) | Abcam, Inc. (MA, USA) | IF(1:100) |
| Goat Anti-Rabbit IgG H&L (FITC) (ab6717) | Abcam, Inc. (MA, USA) | IF(1:100) |
| Rabbit anti-human GAPDH (#5174) | Cell Signaling Technology, Inc. (MA, USA) | WB (1:1000) |
